# Provision, cough efficacy and treatment satisfaction of mechanical insufflation-exsufflation in a large multicenter cohort of patients with amyotrophic lateral sclerosis

**DOI:** 10.1038/s41598-025-91692-8

**Published:** 2025-03-01

**Authors:** André Maier, Dagmar Kettemann, Ute Weyen, Torsten Grehl, Peter Caspar Schulte, Robert Steinbach, Annekathrin Rödiger, Patrick Weydt, Susanne Petri, Joachim Wolf, Julian Grosskreutz, Jan Christoph Koch, Jochen H. Weishaupt, Simone Rosseau, Jenny Norden, Peter Körtvélyessy, Birgit Koch, Teresa Holm, Barbara Hildebrandt, Peggy Schumann, Bertram Walter, Alessio Riitano, Christoph Münch, Thomas Meyer, Susanne Spittel

**Affiliations:** 1 Corporate Member of Freie Universität Berlin and Humboldt-Universität zu Berlin, Outpatient Center for ALS and Other Motor Neuron Diseases, Charité – Universitätsmedizin Berlin, Augustenburger Platz 1, 13353 Berlin, Germany; 2 Center for ALS and Other Motor Neuron Disorders, Berufsgenossenschaftliches Universitätsklinikum Bergmannsheil, Bochum, Germany; 3 Department of Neurology, Center for ALS and Other Motor Neuron Disorders, Alfried Krupp Krankenhaus, Essen, Germany; 4 Clinic for Pneumology, Gastroenterology and Internal Medicine, Alfried Krupp Krankenhaus, Essen, Germany; 5 Department of Neurology, Jena University Hospital, Jena, Germany; 6 Department for Neurodegenerative Disorders and Gerontopsychiatry, Bonn University, Bonn, Germany; 7 Deutsches Zentrum für Neurodegenerative Erkrankungen, Research Site Bonn, Bonn, Germany; 8 Department of Neurology, Hannover Medical School, Hannover, Germany; 9 Department of Neurology, Diako Mannheim, Mannheim, Germany; 10 Precision Neurology, University of Lübeck, Lübeck, Germany; 11 Department of Neurology, University Medical Center Göttingen, Göttingen, Germany; 12 Division for Neurodegenerative Diseases, Neurology Department, Mannheim Center for Translational Medicine, University Medicine Mannheim, Heidelberg University, Mannheim, Germany; 13 Pneumological ventilation center, Ernst von Bergmann Klinik Bad Belzig, Bad Belzig, Germany; 14 Ambulanzpartner Soziotechnologie APST GmbH, Berlin, Germany

**Keywords:** Motor neuron disease, Neurodegenerative diseases

## Abstract

In patients with amyotrophic lateral sclerosis (ALS), mechanical insufflation-exsufflation (MI-E) addresses cough deficiency to achieve major therapeutic goals: improving costal muscle and joint function, reducing atelectasis through insufflation, and clearing bronchial secretions via exsufflation. Despite its perceived benefits, there is limited systematic research on MI-E provision, symptom alleviation, or patient satisfaction. The research platform Ambulanzpartner coordinated this longitudinal observational study conducted in 12 German ALS centers from July 2018 to September 2023. Patients were enrolled based on ALS-related cough deficiency requiring MI-E therapy. The study recorded provision, reasons for withholding MI-E, clinical parameters, therapy frequency, subjective cough deficiency, and symptomatic relief. Satisfaction with MI-E therapy was determined by the likelihood of recommendation. Out of 694 ALS patients indicated for MI-E, 527 (75.9%) received the therapy. The primary reason for non-provision was that the patient had died before provision (n = 66 of 167; 39.5%). These patients were significantly more affected as represented by higher progression rates and lower cough peak flows (CPF) at the time of MI-E indication (*p* < 0.05). Most patients who received MI-E used it daily (n = 290 of 370; 78.4%). Self-assessed cough deficiency correlated with clinical measurements, especially for patients with higher deficits. At follow-up visits, patients reported reduced cough deficiency (*p* < 0.001). Frequent MI-E use was linked to greater symptom relief and higher likelihood of recommending the therapy. This study highlights the symptomatic and palliative potential of MI-E therapy for ALS patients.

**Supplementary Information:**

The online version contains supplementary material available at 10.1038/s41598-025-91692-8.

## Introduction

In amyotrophic lateral sclerosis (ALS) loss of voluntary motor function results in weakness of the tongue, pharyngeal and laryngeal muscles, weakness of the costal, diaphragmatic, abdominal, and accessory muscles^1^. The resulting hypoventilation syndrome leads to respiratory failure, which is the major cause of mortality in ALS^2^. While therapeutic strategies include the provision of non-invasive or invasive ventilation, they are often not technically feasible, not tolerated, or not desired^3^. Beyond hypoventilation, respiratory difficulties decreases reflexive and voluntary coughing abilities and result in a reduction in airway clearance^4,5^, which may lead to atelectasis, acute respiratory failure, and pneumonia^6^. Moreover, with bulbar symptoms the pharyngeal accumulation of excessive saliva may aggravate an already critical condition and lead to distressing choking symptoms. Symptomatic treatment includes drug therapy for saliva management^7^ and techniques to maintain or improve coughing^8–12^.

Mechanical insufflation-exsufflation, an established therapy known as MI-E or “cough assist,” is also increasingly being used and recommended^13^.

MI-E is a cough augmentation strategy in which a device gradually inflates the lungs (insufflation), increases the cough peak flow, mobilizes intercostal muscles groups, and reduces atelectasis^14^. The transition to negative pressure (exsufflation) results in a significant clearance of airway mucus^15^.

The indication of MI-E is based on objective criteria—most often, peak expiratory flow (PEF) or cough peak flow (CPF). Both values differ with respect to the maximum value to be reached in the measurement, since moderately higher values are usually achieved through a coughing maneuver. However, with reduced expiratory force, an increasing convergence of values is evident^16^, meaning that the ability to cough forcefully declines early in the disease course. Although MI-E therapy increases the peak flow and this is generally considered predictive of cough efficiency, this surrogate outcome do not fully capture airway clearance and so the benefits of MI-E therapy for ALS patients remains inconclusive^17,18^. However, use of MI-E can reduce morbidity and need for hospitalization^19^ and prolongs survival when utilized alongside non-invasive ventilation^20^. Case studies and clinical experience support the recommendation to provide MI-E to ALS patients with reduced expiratory peak flow and cough deficiency^21–23^. The initiation of MIE therapy for ALS can be unsuccessful. In particular, due to bulbar involvement, which may cause laryngeal adduction or backward movement of the tongue during insufflation, even at lower insufflation pressures. Also, during exsufflation laryngeal adduction and constriction of the hypopharynx occurs, often irrespective of the presence of bulbar symptoms. A reduced laryngeal response precludes air-filling of the lungs during insufflation, causing discomfort and subsequent inefficient exsufflation^24,25^. Therefore, individualized customized settings of the MI-E are essential, requiring titration starting at low pressure levels during adjustment, considering additional factors such as a well-sealed face mask and specific profiles^11,25^.

Despite the wide application of MI-E therapy, only limited information is available about patients with ALS using MI-E in home care settings^26^. In particular, there is a need to examine the relationship between an objectively measured and perceived cough impairment because this might influence adherence to cough augmentation therapies like the application of MI-E in routine clinical care. Self-perceived cough capacity of people with ALS is studied only to a limited extent and self-reported values may not match the measured values of CPF^27^. To date, very little systematic data has been collected on the effects of MI-E use in ALS patients as viewed through the lens of patient-reported symptom relief^26,28^.

Thus, the aims of the present study are (I) to evaluate the provision rates and causes of failed procurement of MI-E; (II) to determine the frequency of use of MI-E; (III) to analyze subjective cough deficiency in correlation to symptom relief provided by MI-E; and (IV) to identify patients’ satisfaction with MI-E therapy.

## Material and methods

### Study design

A prospective multicenter cohort study was conducted at 12 specialized ALS treatment centers in Germany between 07/2018 and 09/2023. This study was conducted in accordance with the STROBE criteria^29^.

### Participants and inclusion criteria

Subjects who met the following criteria were included in this study: an ALS diagnosis following the revised El Escorial criteria^30^, being at least 18 years of age, a newly diagnosed cough deficiency, and having granted informed consent to participate in the Ambulanzpartner Soziotechnologie (APST) research and case management platform^31–33^. Participants with a severe life-limiting disease other than ALS or with a clinically significant cognitive impairment were not eligible for this trial.

### Ethical approval and consent to participate

The study was conducted in accordance with the established guidelines and regulation. The study was approved by the Medical Ethics Committee of Charité – Universitätsmedizin Berlin, Germany, under code EA1/219/15. Subjects received information about the study both verbally and in writing. Informed consent was obtained from all subjects.

### Setting

#### Diagnosing cough deficiency, initiation of MI-E therapy, and case management

The primary diagnosis of cough deficiency was established by ALS/MND-trained neurologists and experienced nurses in contributing centers through an assessment interview, by measuring cough peak flow (CPF), slow vital capacity (SVC), and by performing a clinical examination. There are generally two conditions for diagnosing a cough deficiency, of which one should be fulfilled: Either the patient showed a value below 270 l/min during CPF screening or a perceived cough deficiency is reported despite formally sufficient cough values, as often occurs in patients with a particularly rapid course of the disease. Following consultations and mutual review with the patient based on shared decision-making principles, an ALS/MND-trained neurologist undertook the final medical indication for MI-E.

Once these criteria were met, the patient was referred to a tertiary respiratory medicine center or, if home assessment was suitable, to a certified respiratory therapist (CRT) by the case management service provided by APST. After verification of the cough deficiency, MI-E therapy initiation was usually supported by a CRT, either in outpatient or inpatient settings. In Germany, CRTs are healthcare professionals who have completed an additional two-year qualification program and receive continuing yearly education. Patients and their relatives received training in the use of the device, while support, accessory, and supply services were established. The adaption procedure of MI-E and recommendations of its use followed latest international literature^34,35^ and the Guidelines for Non-Invasive and Invasive Home Mechanical Ventilation for Treatment of Chronic Respiratory Failure by the German Society of Pneumology and Mechanical Ventilation (DGP)^23^. APST coordinators recorded whether an when treatment was initiated after a patient was given a medical indication for MI-E therapy.

#### Provision and reimbursement of mechanical insufflation-exsufflation (MI-E)

In Germany, in a universal multi-payer health care system the costs of MI-E are usually covered by compulsory health insurances. A smaller percentage of patients are privately insured, but this does not change the cost coverage process. Specialized providers who deal directly with health insurances based on physician prescriptions provided the MI-E devices, service, and training. The approval process with the insurers is usually started directly at the time of MI-E initiation, whereby upfront provision is possible if there is a medical requirement and the providers have made specific arrangement with the insurers. The choice of MI-E model was left to the discretion of the CRT, as long as state-of-the-art technology was used. This involves using new devices or, for cost reasons, devices that have been refurbished from a pool and are in mint condition. The provision process was followed systematically by APST and reasons for the failure to provide MI-E therapy were recorded.

#### Assessment and data capture

All coordinated care and supply data was digitally documented by APST as part of the provision of service. Trained and certified nurses performed respiratory examinations. Neurologists, study coordinators, and study assistants documented clinical and demographic data on distinct case report forms for baseline and follow-up visits. Patient-reported outcomes were captured through printed and digital questionnaires, and structured interviews were conducted via APST’s digital research platform.

### Variables

#### Demographic and clinical data

Collected demographic and clinical data included gender, BMI, age at symptom onset, disease duration, and the findings of the self-administered ALS Functional Rating Scale-Revised (ALSFRS-R)^36^. The ALSFRS-R is a clinically validated and widely used diagnostic instrument that assesses the fine and gross motor functions of arms and legs, bulbar functioning and ventilation. It comprises 12 short questions with 5 anchor points (0–4) as response options. The scale ranges from 0 to 48 points, with the bottom end of the scale reflecting low functionality and more pronounced disease severity. A monthly decline in ALSFRS-R points, or delta ALSFRS-R, is indicative of the rate of deterioration and has prognostic significance^37^. Two items, dysphagia and mechanical ventilation, were used to determine the percentage of enteral nutrition (PEG) and non-invasive ventilation (NIV) at baseline and follow-up.

#### Respiratory data

Data reflecting three respiratory parameters that are routinely measured in ALS specialty centers were included as part of this study. First, the slow vital capacity (SVC) is a standard measurement in ALS. It can determine the degree of neuromuscular hypoventilation syndrome and has high prognostic value^38,39^. The indication of MI-E is based on cough peak flow (CPF) in addition to examined or reported cough weakness. CPF, the second parameter, measures maximal expiratory airflow velocity during a coughing episode and provides predictive value in terms of the patient’s ability to effectively clear their airway of tracheal aspirate during swallowing and salivary secretion^13,27^. Below the threshold values of 270 l/min, ineffective coughing is known to result in a greater risk of respiratory failure and below 240 l/min the incidence of aspiration increases, especially in people with ALS^23,40^. Blood gas parameters such as oxygen saturation correlate with respiratory functioning in ALS patients and also serve as prognostic markers^41^. Oxygen saturation was the third documented parameter.

Assessment methods for these parameters were not specified, and were instead based on standards of care. In most cases, SVC and CPF measurements were taken with hand-held devices, and oxygen saturation was determined with a pulse oximeter.

#### MI-E use

The patient survey recorded the frequency of MI-E use, which was delineated as follows: less than once a day, 1 to 2 times per day, 3 to 4 times per day, and more than 4 times per day.

#### Self-assessment of cough deficiency

A major objective of this study was to elucidate the subjectivity of reduced cough efficiency in relation to the objective parameter CPF. Patients were asked how difficult it was for them to cough effectively both at the time of indication for MI-E (i.e., before adoption) and after the provision of the device. Responses were registered on a Numerical Rating Scale (NRS) ranging from 0 (no difficulty) to 10 (strongest difficulty). To enhance evaluability, the following groupings were made: 0 = no cough deficiency, 1–3 = mild cough deficiency, 4–6 moderate cough deficiency, 7–10 = severe cough deficiency.

#### Subjective assessment and recommendation of MI-E

Following initiation of the therapy, patients were surveyed about MI-E and their level of satisfaction with it. Patients were asked whether the therapy relieved discomfort associated with difficulty coughing effectively, and answers were registered on an NRS with a range of 0 (no relief) to 10 (highest relief).

The Net Promoter Score (NPS) is an easy-to-use instrument that shows moderate to strong correlation with more sophisticated multi-dimensional patient experience and satisfaction questionnaires^42^. It was used in our study to evaluate participants’ attitudes towards MI-E as an indirect measure of satisfaction. Originally developed for customer relationship management, the NPS has become a tool for evaluating medical devices and clinical applications^31,43,44^. For this study, we calculated NPS scores in response to the question: “How likely is it that you would recommend MI-E to another friend or patient with difficulties coughing effectively?”. Based on an 11-point Likert scale ranging from 0 (recommendation absolutely unlikely) to 10 (recommendation highly likely), participants who gave the therapy a 9 or 10 were considered “promoters” (likely to recommend), those who gave it 7 or 8 points were considered "indifferent," and responders who gave the MI-E 6 to 0 points were considered “detractors” (unlikely to recommend). A final score was determined by subtracting the percentage of detractors from the percentage of promoters. In general, a group with a positive NPS is regarded as supportive, and results over 50 are considered excellent^45^. To avoid categorization difficulties, it is also possible to omit the NPS calculus and merely report average values as they correspond to a given recommendation^42^.

### Statistical methods

Data was analyzed with IBM SPSS Statistics (Version 27.0). Scales or subscales for which total or composite scores were included in the statistical analysis (e.g. ALSFRS-R) were only considered in the analysis if items were complete. Consequently, different numbers of patients (n) may be included in the individual result sections. Descriptive analyses were conducted to compare frequencies within assessed parameters. Results were expressed as means (± SD) if distribution was normal, and as medians if numerical data was visualized or if distribution was non-Gaussian. Significant differences between the parameters and, respectively, subgroups of nominally scaled data were assessed by applying contingency tables and Chi-square test. Statistically significant differences of paired samples were analyzed by t-tests. The Wilcoxon test was employed to analyze the statistical power of ordinally-scaled data, while metric data were subjected to the t-test. Correlational analysis was performed with Spearman’s Rho because of the ordinal nature of one of the related scales. To discern group differences within nonparametric data, the Mann–Whitney *U* test was performed on two independent samples. Statistical significance was ascertained with a risk of error of up to 5% (*p* value < 0.05).

## Results

### Sample characteristics, therapy indication, and reasons for failure of therapy initiation

**Fig. 1:**
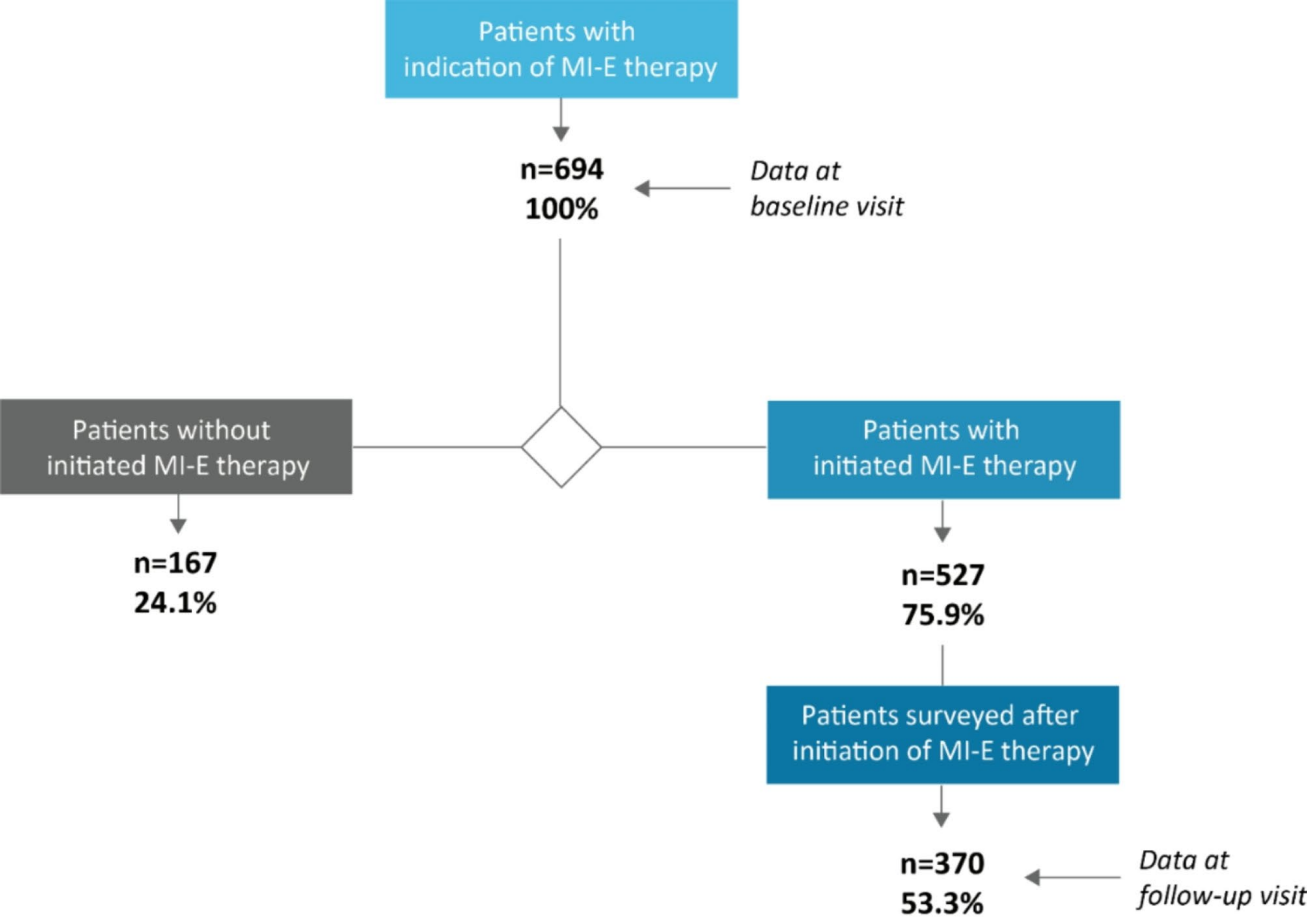
Sample characteristics of studied cohort. n = number of patients.

Within the observation period of 62 months (5 years and two months), an indication for MI-E therapy was given to 694 patients who were subsequently included in this study (baseline visit). In 75.9% (n = 527), MI-E therapy was initiated, and a device was provided. After the indication was given, the latency to initiation had a median of 32 days with a high range from 0 days when the adaption could be implemented immediately in the department prior to reimbursement, to very high latencies up to an extreme value of 268 days if the treatment was severely delayed for various reasons. A second visit to collect follow-up data was carried out after an average of 6.2 months (SD = 5.7 months). The follow-up visit comprised 70.2% (n = 370) of patients originally treated with MI-E (Fig. 1).

**Fig. 2:**
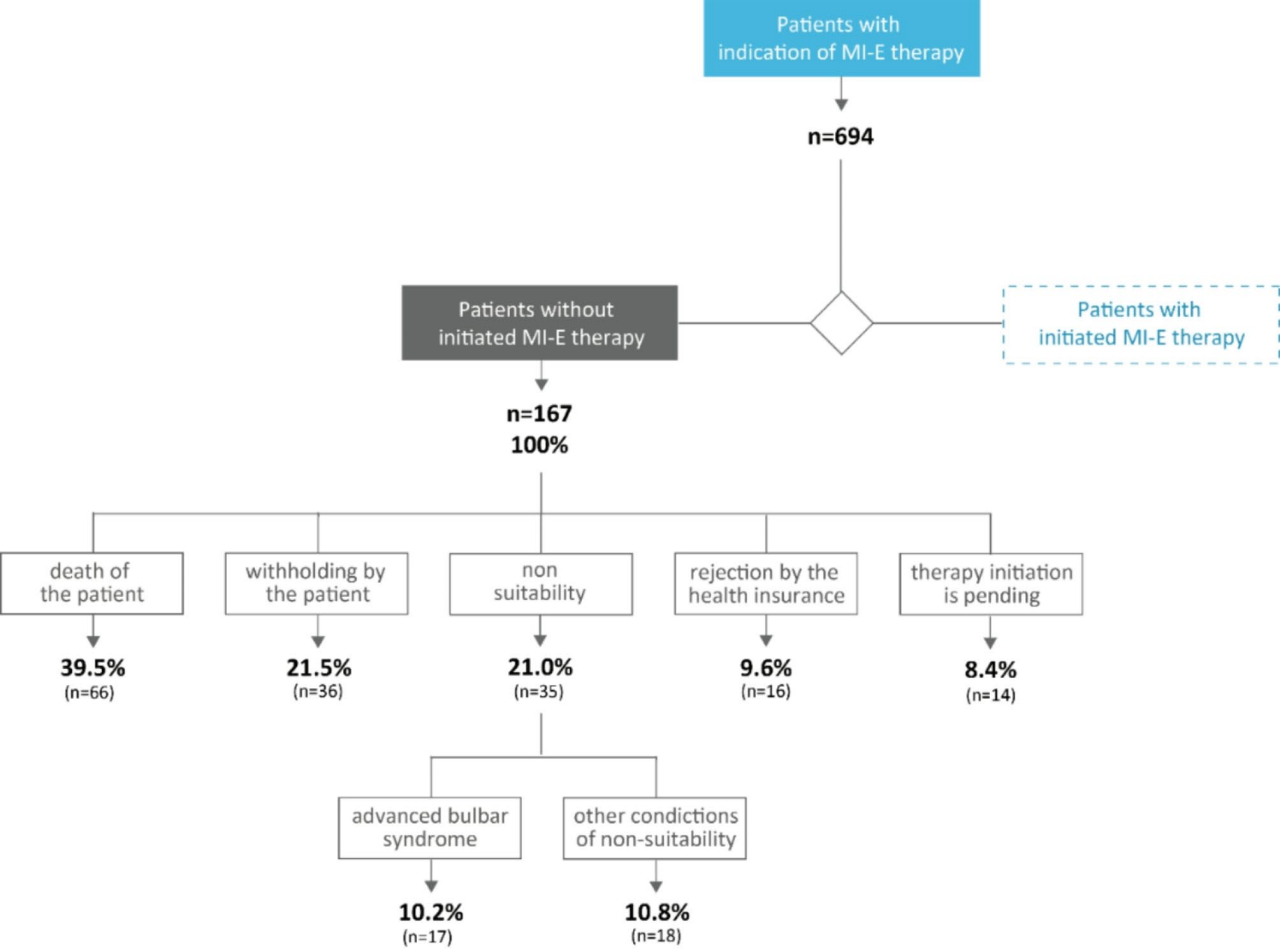
Rate of, and reasons for, non-initiation of MI-E therapy. n = number of patients.

MI-E therapy was not initiated in a total of 167 patients of the total cohort (24.1%). The main reason for failure to receive MI-E therapy was death of the patient prior to provision (39.5%, n = 66 of 167, Fig. 2). Other reasons were patient reluctance, despite having received an indication for MI-E therapy (21.5%, n = 36); medical or practical reasons such as advanced bulbar syndrome, or contraindications such as emphysema, handling, practicality (21.0%, n = 35); and in rare cases, an insurance company rejecting the provision of MI-E therapy (9.6%; n = 16). For 14 patients (8.4%) the initiation of the MI-E therapy was pending at the time of database closure.

### Demographic data and baseline characteristics

The gender distribution (male to female) was 1:1 (49.8% vs. 50.2%), with the study cohort representing a higher proportion of women relative to the general ALS patient population^46^. The age of ALS onset was within the range of prevalence for the general European population of ALS patients (63.7 years, SD 11.1)^47^. There was no significant age difference between men and women, either at symptom onset or at therapy initiation. At the time of MI-E therapy indication, the average disease course was 33.0 months (2.75 years).

Across the total cohort, ALS was moderately advanced, with a mean of 31.4 ALSFRS-R points, but the range was considerable (1–46 points). A decrease in the ALSFRS-R respiratory subscale indicated respiratory involvement (10.3 out of 12, SD = 2.4). Across the entire cohort the disease progressed at an average rate of loss of approximately 0.95 points per month.

With regard to CPF, the clinical measure that supports the indication for MI-E therapy, 93.9% of patients (n = 561 out of 597 with measured CPF) who received the medical indication for the treatment and 93.4% (n = 425 of 455) of those who obtained MI-E were below the established baseline threshold of 270 l/min.

An overview of the demographic and clinical characteristics is provided in Table 1.

**Table 1:** Demographic and clinical characteristics of participants.

Characteristics	Classification	Values
Sex, n = 694	Female, % (n)	50.3 (349)
	Male, % (n)	49.7 (345)
Age, n = 694	At onset, years, mean (SD, R)	63.7 (11.1, 29.4–88.8)
n = 694	At time of initiation of MI-E, years, mean (SD, R)	66.5 (10.7, 30.7–89.8)
n = 580	At time of use of MI-E, years, mean (SD, R)	67.0 (10.7, 32.6–89.8)
Disease duration, n = 694	At time of initiation of MI-E, months, mean (median, IQR, R)	33.0 (20.3, 24.8, 1.9–312.3)
Disease progression, n = 687	Mean (SD, R)	0.95 (0.91, 0.04–9.08)
ALSFRS-R total score (max. 48), n = 687	At time of initiation of MI-E therapy, mean (SD, R)	31.4 (7.5, 1–46)
ALSFRS-R respiratory sub-scale score (max. 12), n = 687	At time of initiation of MI-E therapy, mean (SD, R)	10.3 (2.4, 0–12)
Body Mass Index (BMI), n = 671	kg/m^2^, mean, (SD, R)	24.0 (4.5, 13.5–43.3)
Weight, n = 672	kg, mean (SD, R)	69.7 (14.8, 39–134)
Cough Peak Flow (CPF), n = 597	l/min, mean (SD, R)	163.0 (89.8, 0–417)
Slow Vital Capacity (SVC), n = 631	%, at time of indication of MI-E therapy, mean (SD, R)	65.1 (23.3, 8–150)
Cough deficiency, n = 685	At time of initiation of MI-E therapy (NRS; SD)	6.4 (2.3)
Grouping	No cough deficiency (NRS = 0), % (n)	3.1 (21)
	Mild cough deficiency (NRS = 1–3), % (n)	10.2 (70)
	Moderate cough deficiency (NRS = 4–6), % (n)	26.4 (181)
	Severe cough deficiency (NRS = 7–10), % (n)	60.3 (413)

n, number of participants; SD, standard deviation; R, range; ALSFRS-R, Amyotrophic Lateral Sclerosis Functional Rating Scale, revised; MI-E, mechanical insufflator-exsufflator; IQR, interquartile range.

### Patients with failed initiation of MI-E therapy

Patients who did not receive MI-E treatment (n = 167), the clinical parameters showed a significantly higher rate of disease progression (1.16 vs. 0.89, *p* < 0.01), a higher clinical deficit (ALSFRS-R 30.2 vs. 31.8, *p* < 0.05), and a lower CPF (150 vs. 168, *p* < 0.05). For those patients the age at indication was higher (68.6 vs. 65.8 years, *p* < 0.01), whereas vital capacity was lower without statistical significance (SVC 62.5 vs. 65.9, *p* = 0.117).

The analysis of patients who died before the initiation of MI-E therapy (n = 66) compared to those who did not receive it for other reasons (n = 87) showed a significantly higher general clinical deficit (27.3 vs. 32.3, *p* < 0.001), a higher rate of progression (1.73 vs. 0.74, *p* < 0.001), and lower CPF and SVC (116 vs. 181 and 53.4 vs.69.1, *p* < 0.001) despite patients in both groups being of a similar age (69.9 vs. 68.8).

Subjective baseline cough deficiency, as determined by the NRS, was not significantly different across all groups (MI-E therapy initiated, not initiated, died before initiation: 6.33 vs. 6.46 vs. 6.66).

### Respiratory and clinical follow-up data

Table 2 presents the measured and captured longitudinal outcome data, which were collected at an average interval of 6.2 months (SD 5.7). In particular, it is noticeable that all clinical values showed a significant, albeit expected, progression between the baseline and follow-up visit.

**Table 2:** Respiratory and clinical characteristics before and after initiation of MI-E therapy.

Variable	Number of patients, n	Baseline survey, M (SD)	Follow-up survey, M (SD)	Difference, M (SD, 95% CI)	*p* value^a^	d^b^
ALSFRS-R total score (max. 48)	371	32.1 (7.1)	26.5 (9.0)	5.57 (6.30, 4.92–6.21)	< 0.001	0.884
ALSFRS-R respiratory sub-scale score (max. 12)	369	10.3 (2.5)	8.9 (3.5)	1.42 (2.80, 1.14–1.71)	< 0.001	0.510
CPF in l/min	186	184.3 (84.9)	160.1 (100.3)	24.29 (95.59, 10.46–38.12)	< 0.001	0.254
Cough deficiency	363	6.4 (2.3)	5.7 (2.4)	0.7 (n/a, n/a-n/a)	< 0.001	n/a
SVC in %	196	68.1 (23.3)	55.8 (26.7)	12.25 (17.61, 9.77–14.73)	< 0.001	0.539
SpO^2^	201	95.6 (2.4)	95.0 (2.6)	0.62 (2.92, 0.21–1.02)	< 0.001	0.211
BMI	337	24.2 (4.5)	23.5 (4.5)	0.69 (1.71, 0.51–0.88)	< 0.001	0.405
Weight	342	69.9 (15.1)	68.0 (15.3)	1.81 (5.77, 1.20–2.42)	< 0.001	0.314

^a^Mean difference was accessed by t-test or Wilcoxon signed rank test. A *p* value < 0.05 was considered as statistically significant.

^b^Effect size was classified as follows: low effect size: d ≥ 0.2, medium effect size: d ≥ 0.5 and high effect size: d ≥ 0.8 (Cohens, 1988).

CPF, Cough Peak Flow; SVC, slow vital capacity; SpO, peripheral oxygen saturation; d, effect size; Cohen´s d; M, mean; SD, standard deviation; n, number of patients.

The assessments of the measurements were performed at time of indication for MI-E therapy (baseline survey) and after initiation of MI-E therapy (follow-up survey).

BMI, an important prognostic value for survival in ALS, decreased by 0.7 BMI units or 2.9% (*p* < 0.001). Respiratory parameters of lung functioning (SVC and CPF), which were already on low levels at baseline, showed decreases of 9% and 14%, respectively, at follow-up visits (*p* < 0.001). Interestingly, despite not being clinically meaningful enough at the group level, oxygen saturation values were significantly reduced at the time of the follow-up visit (95.6 to 95.0%; *p* < 0.001).

Analysis of the individual items on the ALSFRS-R reveled that the percentage of enteral nutrition use increased from 7 to 16% in the total cohort from baseline to follow-up. While 11% of the participants used NIV with differing degrees of intensity at baseline, this figure had risen to 31% at the follow-up visit.

### Use of MI-E

At the time of the follow-up visit, a majority of patients were receiving MI-E therapy 1 to 2 times per day (47.0%, n = 174). However, more than a fifth of patients used the device less than once a day, including as needed (21.6%, n = 80). Still 29.5% (n = 109) reported usage of three to four times per day (supplementary Fig. 1).

To enhance comparability, data of patients who used the device ≥ 3 times a day (n = 116) was analyzed alongside those who used it < 3 times a day (n = 254). Patients with higher frequency of use had a significantly higher perceived cough deficiency compared to those with a lower frequency of use (NRS: 6.0 vs. 5.0; *p* = 0.001). With regard to self-reported relief of cough deficiency as a result of MI-E therapy, more frequent use related to greater relief (NRS 6 vs. 7; *p* = 0.002).

### Self-assessment of cough deficiency

Subjective perception of cough deficiency was assessed using an NRS (0–10). With the aforementioned grouping, it is evident that the majority of patients reported severe (NRS = 7–10; 60.3%, n = 413) or moderate cough deficiency (NRS = 4–6; 26.4%, n = 181) (supplementary Fig. 2). The mean score at baseline was 6.4 (SD 2.3; n = 685), which is considered moderate to severe.

Among the patients who did not report cough deficiency in their self-assessments, 93.3% (n = 14/15) had a CPF of less than 270 l/min. Conversely, 97.2% of patients who did not present with a formal CPF-value-based indication for MI-E reported a subjective cough deficiency (n = 35/36). The overall correlation between experienced and objectively measured cough deficiency is significant (*p* < 0.001). The correlation coefficient of only 0.25 results from a high scattering, however, indicating a limited correspondence between self-reported and measured deficiency (supplementary Fig. 3).

**Fig. 3:**
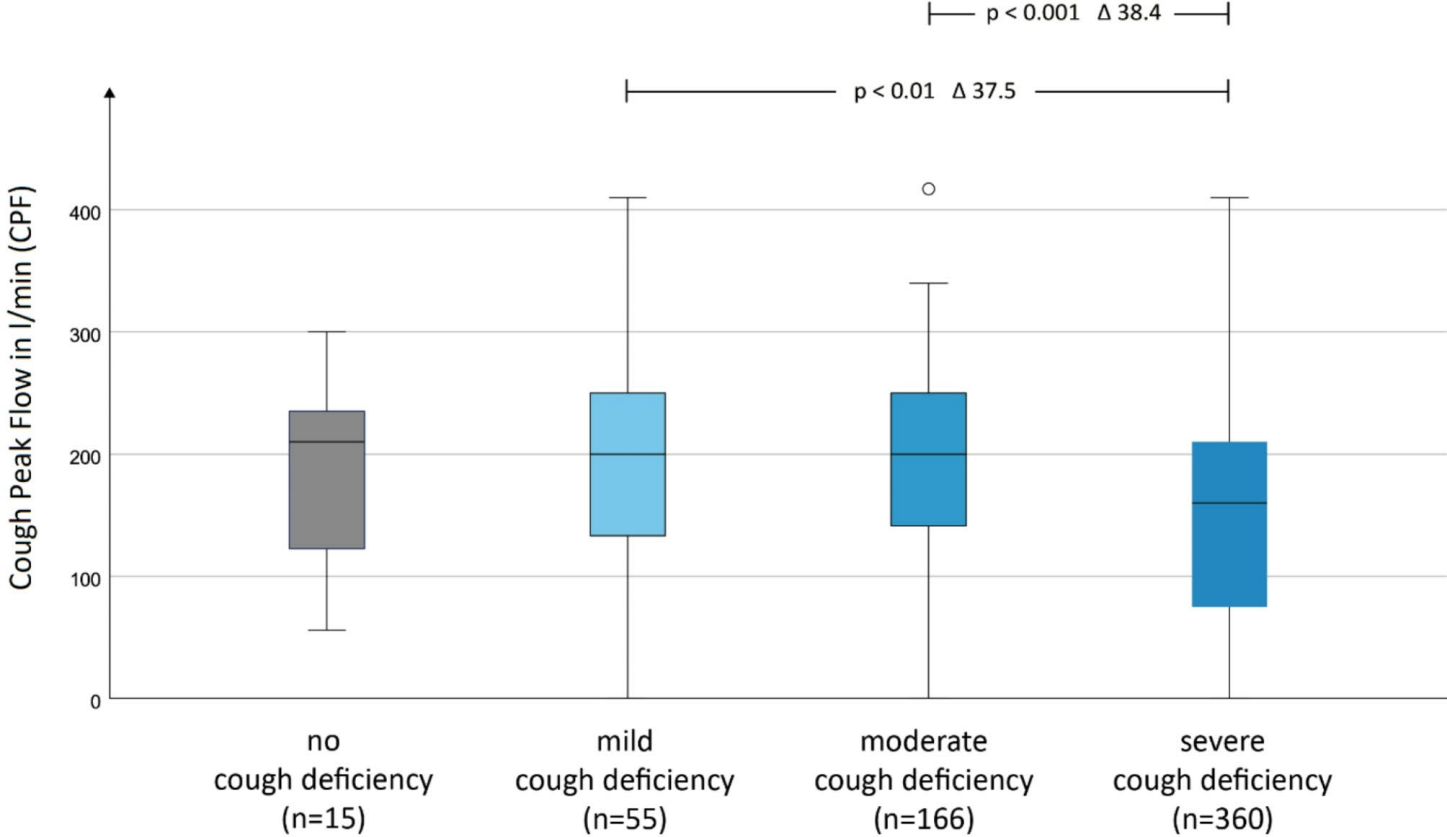
Cough deficiency in relation to the CPF. Cough deficiency was assessed by the Numerical Rating Scale (NRS) ranging from 0 (no difficulty) to 10 (strongest difficulty). To enhance evaluability, groupings were made as follows: 0 = no cough deficiency, 1–3 = mild cough deficiency, 4–6 moderate cough deficiency, 7–10 = severe cough deficiency. n = 596.

A group analysis based on the self-assessment of the deficit revealed a significant difference in CPF between participants reporting a strong deficit and those reporting a moderate or mild deficit (Fig. 3). Such an association cannot be established for no deficit. Overall, the relationship between self-reported and measured deficits is strongest at the higher levels. Self-reported cough deficiency did not depend on differing levels of bulbar involvement, age or gender.

**Fig. 4:**
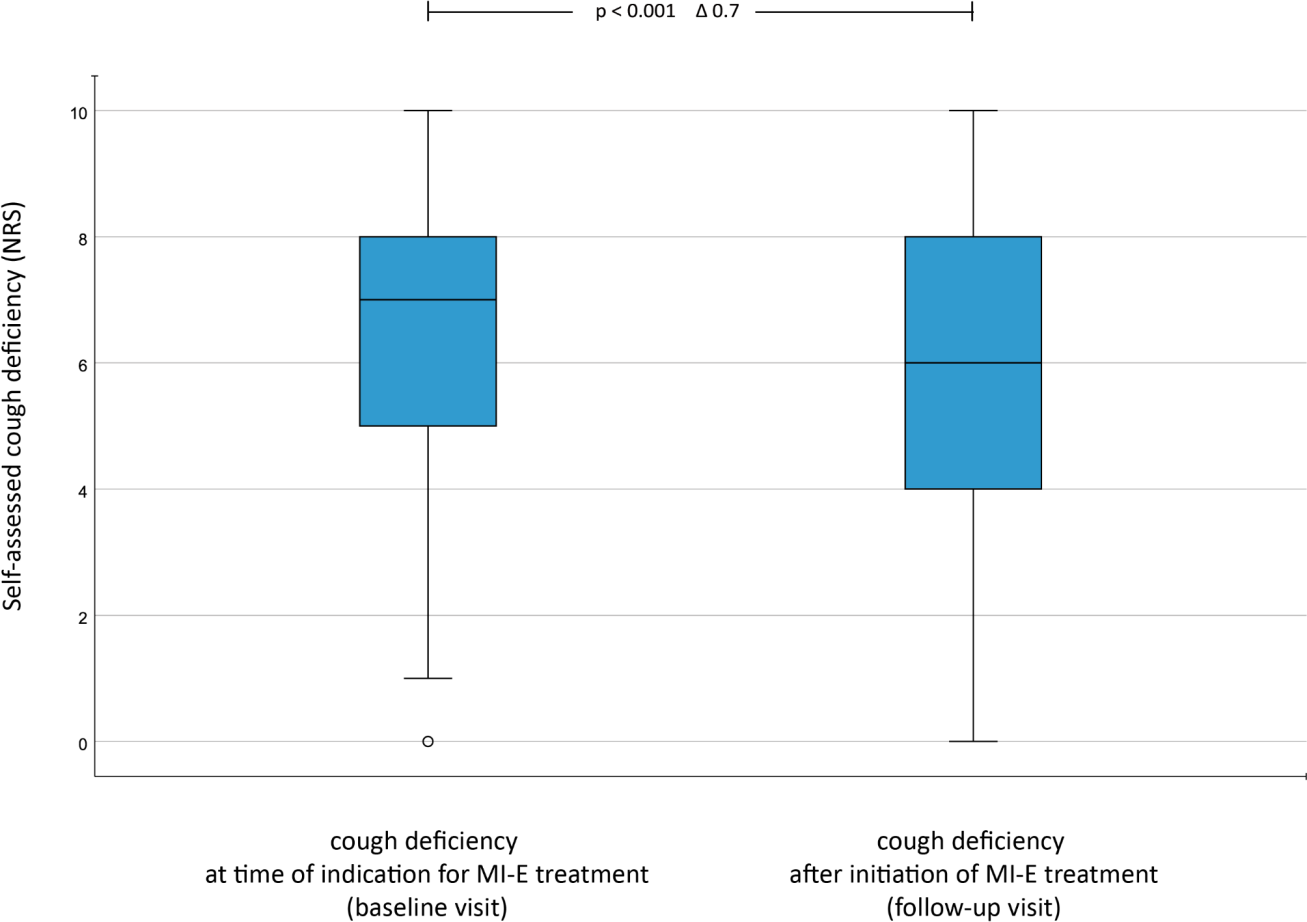
Self-assessed cough deficiency before and after initiation of MI-E therapy. Cough deficiency was assessed by a Numerical Rating Scale (NRS) ranging from 0 (no difficulty) to 10 (strongest difficulty). To enhance evaluability, groupings were made as follows: 0 = no cough deficiency, 1–3 = mild cough deficiency, 4–6 moderate cough deficiency, 7–10 = severe cough deficiency. The interval between baseline and follow-up visits was 6.2 months on average (SD = 5.7). n = 363.

At the time of a follow-up visit 6.2 months (SD 5.74) after the initial visit, respondents rated subjective cough deficiency significantly better than they did at baseline (6.2 vs. 5.7 mean scale points, *p* < 0.001, Fig. 4). The correlation between CPF and self-assessed cough deficiency was comparable between the first and the follow-up visit (r = 0.29).

### Subjective assessment of symptom relief and recommendation of MI-E

When asked about the relief provided by MI-E therapy, more than half of all patients reported that it was strong (51.1%, n = 185) and only 8.0% of all respondents reported having received no relief. 40.9% of patients (n = 148) reported minor to medium symptom relief (supplementary Fig. 4). The group that reported higher levels of relief also showed higher daily use. By separating the participants at the median of an NRS value of 7 (n = 176 vs. 183), higher baseline levels of cough deficiency were found in patients who reported higher levels of relief (p = 0.02). However, disease progression, duration, severity, age, and respiratory parameters did not differ significantly.

**Fig. 5:**
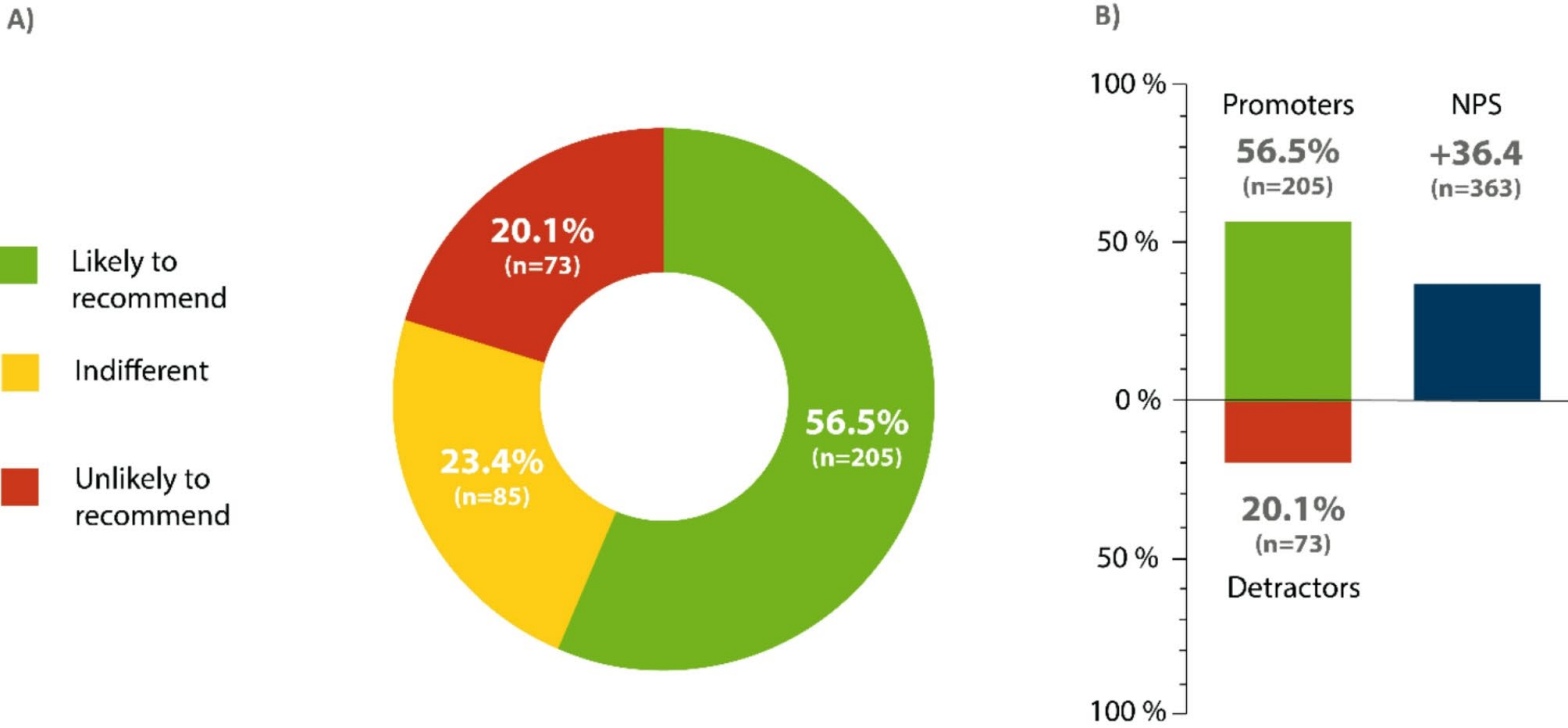
Likelihood of recommending a mechanical insufflation-exsufflation (MI-E). The NPS was used to assess participants’ likelihood of recommending the MI-E. Scores were calculated based on responses to a single question: “How likely is it that you would recommend the MI-E to another friend or patient who is affected with ALS?” Answers ranged between 0 (absolutely unlikely to recommend) and 10 (very likely to recommend). Participants who responded with a score of 9 or 10 were considered “promoters.” Those who gave the therapy a 7 or 8 were classified as “indifferent,” and participants whose rankings were between 0 and 6 were defined as “detractors” (**A**). The NPS was calculated by subtracting the percentage of detractors from the percentage of promoters (**B**). n = 363; n, number of participant.

**Fig. 6:**
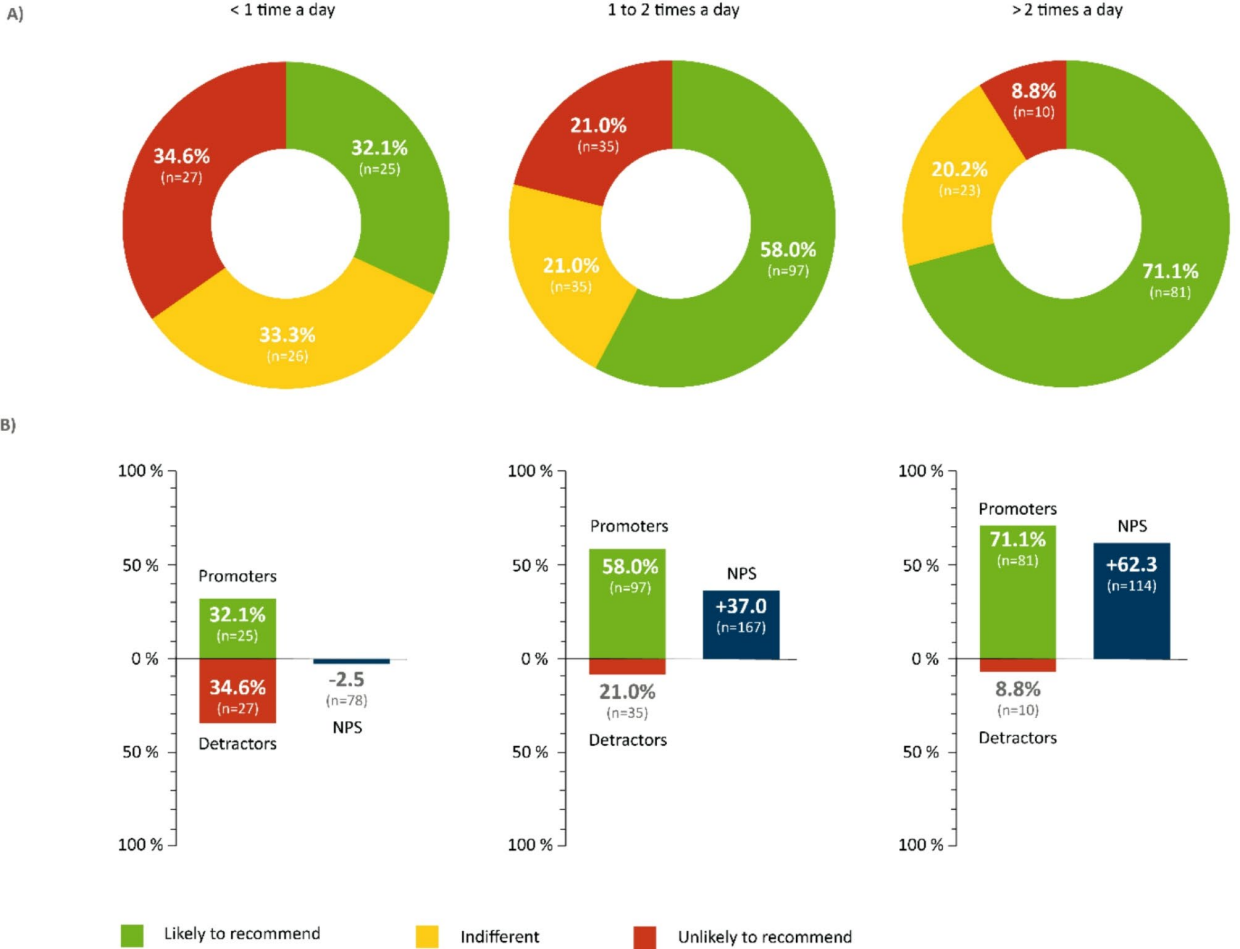
Likelihood of recommending a mechanical insufflation-exsufflation (MI-E) with respect to frequency of MI-E use. The NPS was used to assess participants’ likelihood of recommending the MI-E. Scores were calculated based on responses to a single question: “How likely is it that you would recommend the MI-E to another friend or patient who is affected with ALS?” Answers ranged between 0 (absolutely unlikely to recommend) and 10 (very likely to recommend). Participants who responded with a score of 9 or 10 were considered “promoters.” Those who gave the therapy a 7 or 8 were classified as “indifferent,” and participants whose rankings were between 0 and 6 were defined as “detractors” (**A**). The NPS was calculated by subtracting the percentage of detractors from the percentage of promoters (**B**). n = 363; n, number of participants.

Thus, overall satisfaction with MI-E therapy is high. 56.5% strongly recommended the therapy (9 or 10 points). 23.4% were indifferent (7 to 8 points) and 20.1% gave a weak to no recommendation (0 to 6 points). The NPS was + 36.4 (Fig. 5). A significant correlation was found between frequency of use and the likelihood of recommendation, meaning more frequent MI-E use increased the likelihood of a recommendation (Fig. 6).

The relationship between a patient’s subjective relief for cough deficiency and their likelihood of recommending MI-E was also very strong, with a correlation coefficient of 0.6. Groups reporting no relief, minor relief, medium relief and strong relief showed significantly different recommendation and achieved an NPS value of -65.5, -19.2, + 21.3 and + 76.7, respectively (supplementary Fig. 5).

Women were more likely to recommend MI-E therapy than men, although not by a significant margin (NPS: + 41.6 vs. + 30.9; p = 0.057). Age of respondents did not have impact on recommendation rates. Disease severity, as measured by the ALSFRS-R, had a slight but not significant impact on recommendation rates for MI-E therapy (ALSFRS-R groups subdivided by mean value: ≤ 27 vs. > 27: NPS + 33.1 vs. + 39.8; p = 0.129).

## Discussion

### Sample characteristics, demographic data, and reasons for failure of therapy initiation

Among ALS patients with defined cough deficiency, mechanical insufflation-exsufflation therapy (MI-E) is part of standard care in Germany^21^ and in many other countries to increase airway clearance and reduce the risk of life-threatening conditions such as pneumonia and acute respiratory insufficiency.

Involving 12 centers and 694 patients, this is the largest study to date aimed at investigating the effects of MI-E therapy on people with ALS in a clinical setting. Because of the substantial number of participants, this study was able to reflect a general yet older ALS population at an advanced stage where cough deficiency becomes an important therapeutic target.

The gender ratio within the cohort presenting with an indication for MI-E is unusual, as men are generally more affected than women (1.5:1). This might be explained by the fact that cohorts of older age from population-based studies (> 65 years) report a nearly equal gender ratio^48^ and the cohort is selected by a MI-E indication. The complex interactions between gender, different respiratory involvement of clinical phenotypes, disease progression, and survival^49^ may have also shifted the gender distribution.

Given that MI-E therapy is usually indicated when reduced cough efficacy occurs in the context of respiratory insufficiency, clinical parameters revealed a study population with a more advanced stage of ALS on average, although with a wide range. Already at the beginning of the study, all relevant respiratory parameters as well as the respiratory subscale of the ALSFRS-R and the self-assessment of cough deficiency showed relevant impairment, justifying MI-E therapy. The difference in baseline CPF levels between those who received MI-E treatment and those who died prior to receiving MI-E therapy emphasizes the prognostic value of the metric. Overall, it emerged that provision of MI-E was unsuccessful in nearly a quarter of all cases. ALS-related reasons, including death prior to provision, accounted for around 60% of this figure. A provision latency of a median of 32 days is most certainly a contributing factor. In addition, the specific and complex adaptation of MI-E in ALS also partly provides an explanation for these results. Recent research shows that when using MI-E, about 50% of ALS patients exhibit an obstruction pattern during insufflation, mostly, but not exclusively related to bulbar involvement^34^. Recommendations exist to address this challenge in a reasonable manner, which primarily include individualized titration^35^ and, e.g., precise interpretation of the flow graphs during adaptation^34^. A further 40% of unsuccessful provision had to do with individual reasons relating to the patient, the health insurance provider, or the ongoing provision process.

In particular, patients who were not provided MI-E had higher rates of progression, were older and had a higher cough deficiency. The correlation was even clearer in patients who died before therapy was initiated. It can therefore be concluded that members of this group should have received the indication earlier in the disease process or that the provision process itself needs to be accelerated, e.g. through prioritization.

### Respiratory and clinical follow-up data

Data collection was carried out over two visits with an average interval of 6 months between them. Incomplete clinical data is partly due to the fact that data was recorded during standard procedures. The COVID-19 pandemic in particular led to restrictions in ALS departments regarding the collection of respiratory parameters. As a result, CPF and SVC values were obtained in just over half of follow-up visits. However, the data is sufficient to show that all respiratory parameters decreased significantly with a small to medium effect size. Surprisingly, and although within a marginally acceptable range on average, even standard oximetry showed a significant decline in the cohort. The same applies to the BMI, an important prognostic metric for ALS survival. Although BMI decreased only by 0.7 units, or 2.9%, this represents a 2% reduction in an estimated survival ratio^50^.

Longitudinal data shows an increase in respiratory dysfunction, weight loss and loss of general function on the ALSFRS-R—all parameters that indicate ALS progression.

The evaluation of individual items of the ALSFRS-R showed that the proportion of participants with ventilation support increased from 11% to nearly one third. This indicates that both therapies, MI-E and NIV, are frequently administered together, as improved survival suggests by offering a combined therapy^20^. However, it also demonstrates that the use of MI-E is considerably more prevalent than NIV, which may be due to the lower effort involved and leaves room for improvement. In addition, there is still insufficient evidence on the effects of MI-E on survival in ALS.

### Use of MI-E therapy and self-assessment of cough efficiency

MI-E use mostly followed a therapeutic recommendation to use the device every day or several times a day, although there is no specific consensus about frequency of use^51^. Almost 22% of patients used the device less than once per day (21.6%, n = 80), which reflects use when needed. These numbers correspond to an earlier analysis, where up to 73% used the device on a daily basis^52^. Previous studies have shown a solid correlation between reported and actual usage based on digitally-retrieved data, and found that frequency of use is related to secretion burden^26^. This indicates that adherence is not compromised if use is based on needs arising from respiratory impairment or the stage of disease, as opposed to a pre-determined frequency. Furthermore, a mandate daily use in stable patients is questioned^53^.

At baseline, an overwhelming majority of participants self-reported a cough deficit, with most documenting relatively severe impairment. The strongest relations between self-perception and measured value were in the higher deficit range. However, analysis shows that a threshold value of less than 270 l/min reflects the experienced difficulties of those affected reasonably well, even though some people may notice a deficit even before reaching this value or will underestimate the objective deficit. Yet, a treatment decision should not be based on strict values, but should be considered on an individual basis and maybe even before a patient reaches a CPF of 270 l/min. Interestingly, we found no association between frequency of use and CPF or SVC. Patients with a higher frequency of use show a higher subjective cough deficit but also greater symptomatic relief. From this, it must be concluded that the subjective dimension has a relevant impact on the administration of the therapy.

At follow-up visits, contrary to the clinical parameters, which all decreased, the values of self-assessed cough deficits were significantly better. The question gauging a patient’s cough deficit was deliberately phrased in general terms and did not directly address MI-E therapy. It thus remains an open question whether coughing was perceived to be improved as a result of exertion and reduced secretion accumulation or by better clearance following use of the device.

### Treatment recommendations and perception of MI-E therapy

Regarding cough deficit relief, over 90% of patients reported benefits that most users rated as moderate to strong. It should be emphasized that even more than 50% of all patients described strong relief. In contrast to the subjective assessment of cough deficiency, which is associated with greater relief, clinical parameters such as CPF, SVC, and bulbar score had no statistically significant influence on reported relief.

The overall recommendation for MI-E therapy was high, reflecting high satisfaction. The NPS, which was originally designed for consumer use before it found its way into medicine^42^, shows very good values for all patient groups. Recommendation measured with the NRS and thus the NPS show a high correlation between frequency of use and cough deficiency relief. Frequent users show excellent NPS and significantly greater subjective benefits from MI-E.

### Limitations

This was designed as an observational study which takes possible bias and confounding factors into account. Objective measurements were routinely gathered by specialized ALS centers, although not via highly standardized procedures. Respiratory parameters were therefore regarded as disease progression indicators and guidance values for treatment decisions. The study did not set out to deduce the effects of direct treatment from respiratory measurements. However, different data sources and examination techniques can lead to bias. In addition, the COVID-19 pandemic led to missing data, and in particular, respiratory one. In addition, the follow-up and surveillance of patients may have been affected also by the pandemic.

Individual self-assessment of cough deficit or actual cough strength can be highly subjective and situation-specific. The clinical definition of a cough is complex^54^, and there is certainly no single conception of it. The extent of secretion, pharyngeal obstruction, laryngeal weakness, possible transient infections and psychological factors can all play important roles in the perception of cough efficacy, and may therefore confound assessments.

There are hardly any studies on the self-perception of coughing among ALS patients. Coughing itself, e.g., in public, can have negative social connotations^55^, but it is unclear how and when ALS patients perceive insufficient coughing as a problem. Addressing the issue of coughing on its own before providing MI-E has a desirable educational effect, but can lead to bias as the type and amount of information patients receive can vary considerably.

Patient selection was based on individual indications for MI-E therapy, actual supply of devices, and willingness to participate in the study. While this is implicative of a selection bias and constitutes a limitation, the sample size of the study is such that the actual impacts of these factors are not all that strong.

Certainly, a controlled study that longitudinally considers patients who do not receive MI-E therapy could potentially produce stronger evidence regarding certain measurements of treatment success. Patients who did not receive MI-E did not have their cough efficacy tracked. Given the existing realities of care and the limitations of our approach, this was simply not achievable.

With regard to the strength of the results in terms of cough deficiency and MI-E relief, we must differentiate between self-evaluations performed by patients and evaluations made by medical professionals.

It was the purpose of this study to focus on subjective experiences, i.e., on the fine interplay between a severe, potentially life-threatening symptom and patients’ personal treatment experiences and opinions, as well as their subsequent willingness to recommend the therapy and conclude satisfaction accordingly. Such perceived benefits cannot always be captured by strictly objective measurement techniques. In addition, the brevity of the NPS as an indirect measure of satisfaction is both a strength and a potential source of limitation. In a broad study, a single-item questionnaire allows for a large number of participants, however, there are questions on the extent to which the NPS corresponds to more complex patient experience and satisfaction measures^42^.

Although definitive medical criteria for diagnosing cough deficiency was applied in this study, the questionnaire used to measure this symptom and levels of relief has not been validated. In fact, we relied upon the subjective reports of patients to assess the effectiveness of the therapy. Further studies are needed to medically define and measure the effects of MI-E and to align those results with subjective experience.

## Conclusion

This multicenter study to evaluate mechanical insufflator-exsufflator therapy use was conducted in a real-world clinical care setting. By looking at provision data, it is evident that the MI-E indication largely applies to the intended therapeutic target group within the required scope. Some patients could not be treated due to possibly avoidable obstacles. In particular, data for a small number of patients suggests that a medical indication should have been given sooner, when the disease was less advanced. To enable earlier intervention, process optimization such as progression-rate based or predictive provisioning should be implemented^56^. In addition, immediate home adaptation may provide time benefits prior to admission to a tertiary respiratory center. Innovative screening approaches could be explored in the future, such as using sensor technology to recording coughing sounds, for example^57^. Given the complexity of MI-E adaption in ALS and the potential variability in the provision and use of MI-E in different ALS populations, there is a need for more standardized international clinical guidelines.

In summary, this study provides a favorable picture of MI-E therapy and its apparent value to patients. With the use of MI-E, cough deficiency is perceived to be lower. Meanwhile, the more frequently the therapy is used, the greater the cough deficiency relief and the higher the likelihood of recommendation. Further studies are needed to determine the impact on survival, e.g. by preventing pneumonia, and – in patients who are more likely to require palliative treatment – on quality of life.

## Electronic supplementary material

Below is the link to the electronic supplementary material.

Supplementary Material 1

## Data Availability

Data for this study was provided by Ambulanzpartner Soziotechnologie APST GmbH. However, because this data was used under license, it is not publicly available. It can be obtained from the author Susanne Spittel (s.spittel@ambulanzpartner.de) upon reasonable request and with the permission of Ambulanzpartner Soziotechnologie APST GmbH: https://www.ambulanzpartner.de/.

## Abbreviations

MI-E: Mechanical insufflation-exsufflation
SVC: Slow vital capacity
CPF: Cough peak flow
NRS: Numeric rating scale
NPS: Net Promoter Score
ALSFRS-R: ALS functional rating scale - revised
CRT: Certified respiratory therapist
