# Supplementary material for: Provision, cough efficacy and treatment satisfaction of mechanical insufflation-exsufflation in a large multicenter cohort of patients with amyotrophic lateral sclerosis
Source: Sci Rep. 2025 Mar 1;15:7360. doi: 10.1038/s41598-025-91692-8 (PMC11873142; doi:10.1038/s41598-025-91692-8)
Supplement: Supplementary file 1 — Supplementary Material 1 [file 41598_2025_91692_MOESM1_ESM.pdf]

Supplementary information:

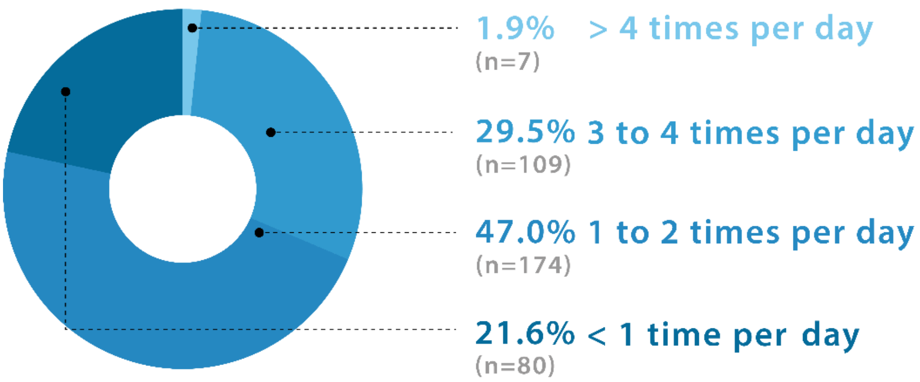

Supplementary figure 1: Use of mechanical insufflation-exsufflation (MI-E).

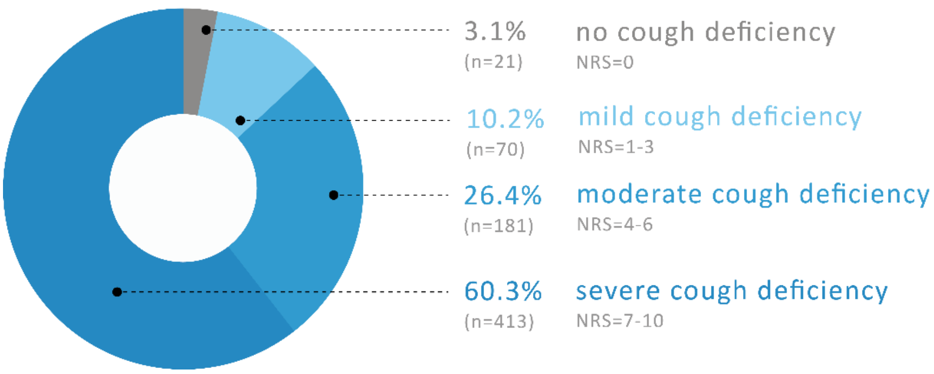

Supplementary figure 2: Self-assessment of cough efficiency at time of indication for MI-E therapy (baseline visit).

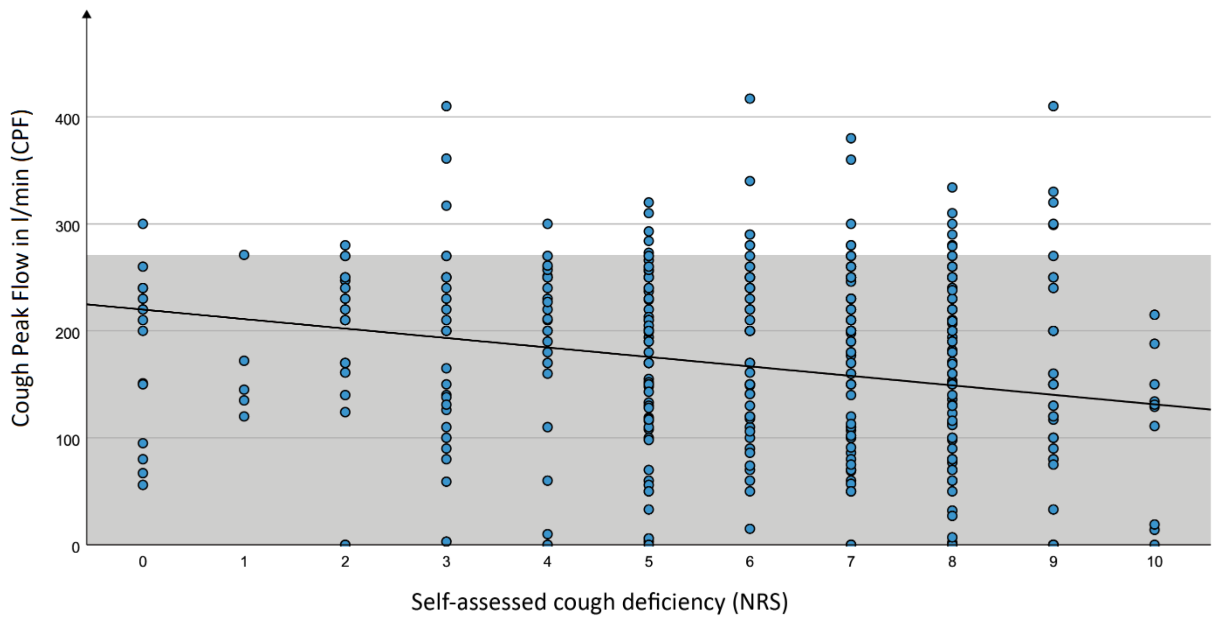

Supplementary Figure 3: Cough efficiency was assessed by the Numerical Rating Scale (NRS) ranging from 0 (no difficulty) to 10 (strongest difficulty). n=596.

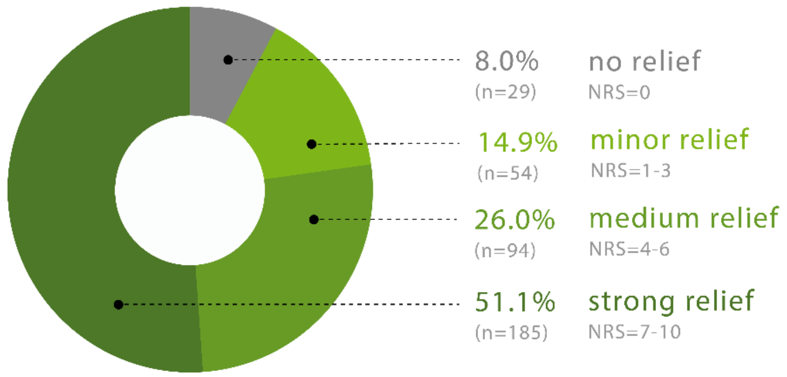

Supplementary figure 4: Relief of cough deficiency through MI-E therapy. Relief through therapy was assessed by the Numerical Rating Scale (NRS) ranging from 0 (no difficulty) to 10 (strongest difficulty). To enhance evaluability, groupings were made as follows: 0 = no cough relief, 1-3 = minor relief, 4-6 medium relief, 7-10 = strong relief, n = 362)

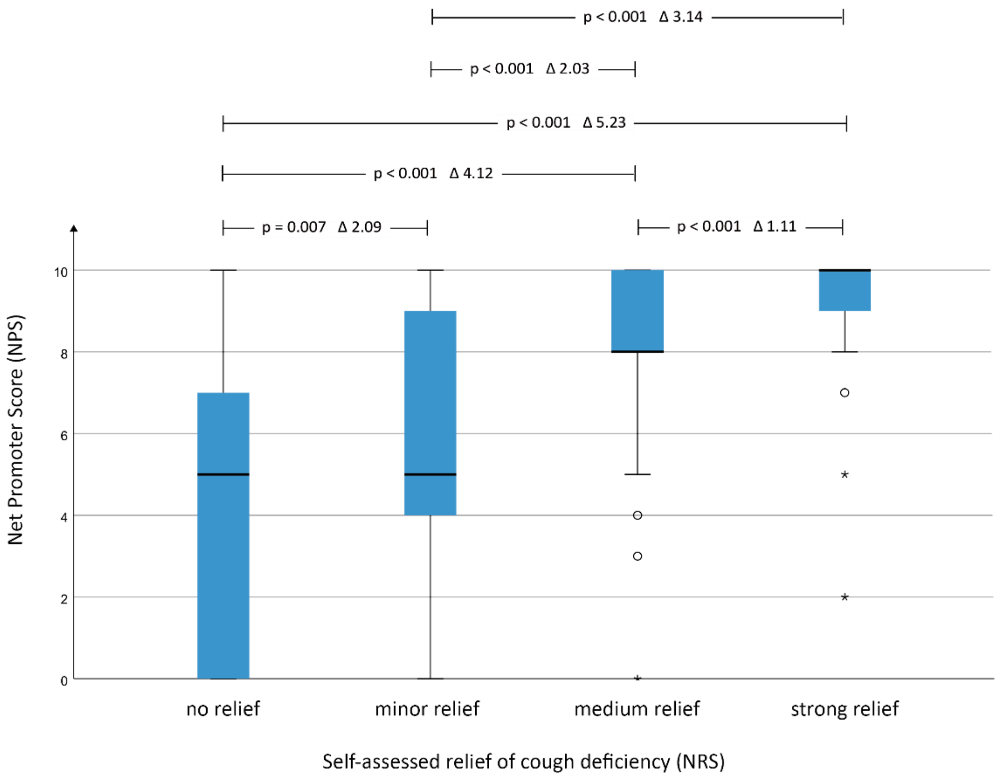

Supplementary Figure 5: Relief of cough deficiency in relation to recommendation MI-E. Relief through therapy was assessed by the Numerical Rating Scale (NRS) ranging from 0 (no difficulty) to 10 (strongest difficulty). To enhance evaluability, groupings were made as follows: 0 = no cough deficiency, 1-3 = mild cough deficiency, 4-6 moderate cough deficiency, 7-10 = severe cough deficiency, n = 359)
